# 3D Printing Continuous Fiber Reinforced Polymers: A Review of Material Selection, Process, and Mechanics-Function Integration for Targeted Applications

**DOI:** 10.3390/polym17121601

**Published:** 2025-06-09

**Authors:** Haoyuan Zheng, Shaowei Zhu, Liming Chen, Lianchao Wang, Hanbo Zhang, Peixu Wang, Kefan Sun, Haorui Wang, Chengtao Liu

**Affiliations:** 1 College of Aerospace Engineering, Chongqing University, Chongqing 400030, China; zhenghaoyuan0125@163.com (H.Z.);; 2 State Key Laboratory of Coal Mine Disaster Dynamics and Control, Chongqing University, Chongqing 400030, China; 3 IMDEA Materials Institute, 28906 Madrid, Spain; lianchao.wang@imdea.org

**Keywords:** 3D printing, continuous fiber reinforced polymers, material selection, process, mechanical properties, functional applications

## Abstract

In recent years, the rapid development of three-dimensional (3D)-printed continuous fiber-reinforced polymer (CFRP) technology has provided novel strategies for customized manufacturing of high-performance composites. This review systematically summarizes research advancements in material systems, processing methods, mechanical performance regulation, and functional applications of this technology. Material-wise, the analysis focuses on the performance characteristics and application scenarios of carbon fibers, glass fibers, and natural fibers, alongside discussions on the processing behaviors of thermoplastic matrices such as polyetheretherketone (PEEK). At the process level, the advantages and limitations of fused deposition modeling (FDM) and photopolymerization techniques are compared, with emphasis on their impact on fiber–matrix interfaces. The review further examines the regulatory mechanisms of fiber orientation, volume fraction, and other parameters on mechanical properties, as well as implementation pathways for functional designs, such as electrical conductivity and self-sensing capabilities. Application case studies in aerospace lightweight structures and automotive energy-absorbing components are comprehensively analyzed. Current challenges are highlighted, and future directions proposed, including artificial intelligence (AI)-driven process optimization and multi-material hybrid manufacturing. This review aims to provide a comprehensive assessment of the current achievements in 3D printing CFRP technology and a forward-looking analysis of existing challenges, offering a systematic reference for accelerating the transformation of 3D printing CFRP technology from laboratory research to industrial-scale implementation.

## 1. Introduction

Three-dimensional printing (3D printing) has evolved from its initial application as a rapid prototyping technology to a manufacturing method capable of producing functional end-use components, fundamentally transforming traditional paradigms in design and production. This technique constructs complex geometries through layer-by-layer material deposition, offering unprecedented design freedom and manufacturing flexibility across diverse fields including aerospace, automotive, biomedical, and consumer goods industries.

Among various materials compatible with 3D printing, polymers have gained significant attention due to their ease of processing and material versatility. However, the mechanical properties of pure polymer-printed parts often prove inadequate for high-performance applications, exhibiting notably inferior strength and stiffness when compared to conventional injection-molded or machined components. To address this limitation, the incorporation of continuous fibers into polymer matrices to form Continuous Fiber Reinforced Composites (CFRCs) has demonstrated substantial enhancement in the mechanical performance of printed parts. This advancement reveals considerable potential for structural applications requiring high strength-to-weight ratios [1,2].

The application of CFRPs in 3D printing represents a significant advancement in additive manufacturing. Compared to short-fiber or particulate reinforcements, continuous fibers stand out due to their ability to align along specific orientations and provide superior load-bearing capabilities [3,4]. This characteristic enables 3D-printed CFRCs to not only meet lightweight and high-strength requirements but also achieve performance customization through precise control of fiber orientation [5,6]. Traditional composite manufacturing methods, such as hand lay-up or automated fiber placement, can produce high-performance components but are often constrained by geometric complexity and the need for expensive molds. In contrast, 3D printing technology offers a novel manufacturing pathway for complex-shaped high-performance composites through mold-free fabrication and efficient material utilization [2,4].

The historical development of 3D-printed CFPRs traces back to the early conceptualization of fiber-reinforced composites. As early as the mid-20th century, the emergence of glass and carbon fiber composites laid the foundation for high-performance applications, with these materials being widely adopted in aerospace and other industries due to their exceptional strength-to-weight ratios [7]. However, limitations in conventional manufacturing processes prompted researchers to explore novel fabrication approaches. Since its inception in the 1980s, 3D printing technology was initially primarily employed for polymer prototyping, but advancements in equipment and material technologies gradually expanded its applications to functional component production. The integration of continuous fibers has further propelled this technology into the realm of high-performance composite materials [1].

The synergistic relationship between material selection and manufacturing processes constitutes the core logic for achieving mechanics-function integrated design in 3D-printed continuous fiber polymer technology. The combination of fiber types and matrix materials not only determines the intrinsic performance boundaries of composites in terms of strength, stiffness, and environmental resistance, but also governs functional behaviors via precise control of interfacial characteristics and microstructure. Process control and parameter optimization determine the feasibility and effectiveness of 3D printing implementations. The coupling among material–process–performance relationships remains the critical focus for advancing CFRP 3D printing technology.

This review will discuss the technological evolution and application potential in target fields such as aerospace lightweight load-bearing structures and biomedical dynamic implants, based on analysis of the recent decade’s literature from material–process–performance perspectives.

The paper is organized as follows: Section 2 provides an in-depth analysis of carbon, glass, and natural fibers’ properties, advantages/disadvantages, and their performance implications. Section 3 focuses on comparative assessments of Fused Deposition Modeling (FDM), vat photopolymerization, and emerging technologies, with emphasis on process parameter effects. Section 4 and Section 5 systematically examine how fiber type, orientation, and post-processing influence mechanical and functional performance of composite. Section 6 evaluates implementation potential through case studies including aerospace components and biomedical implants. Ultimately, this review aims to establish a comprehensive reference framework to facilitate the transition of 3D-printed continuous fiber polymer technology from laboratory research to industrial applications.

## 2. Material Selection

The performance of 3D-printed CFRPs is largely determined by the selection of fibers and matrices. These materials must be chosen based not only on their intrinsic properties but also on their compatibility with 3D printing processes and each other. This section provides a detailed analysis of commonly used fibers, matrix types, their characteristics, and associated selection challenges in 3D-printed CFRCs. A thorough understanding of material properties offers theoretical foundations and practical guidance for optimizing composite performance.

### 2.1. Selection of Fibers

Fibers can be classified into three major categories based on their origin and composition: synthetic fibers, non-synthetic inorganic fibers, and natural fibers. Synthetic fibers such as aramid fibers typically exhibit excellent mechanical properties and heat resistance. Non-synthetic inorganic fibers, primarily including carbon fibers and glass fibers, possess high strength and high modulus, making them widely applicable in high-performance composites. Natural fibers like flax and kenaf have gained increasing attention in sustainable materials research due to their renewability and low density.

Continuous fibers serve as the principal load-bearing elements in CFRCs, delivering exceptional strength and stiffness. As shown in Table 1, carbon, glass, aramid (e.g., Kevlar), and natural fibers are the most widely utilized reinforcement materials.

Carbon fibers dominate high-performance applications like aerospace and automotive industries due to their superior strength-to-weight ratios (3500–7000 MPa tensile strength), high moduli (230–600 GPa), and corrosion resistance [8,9,10,11,12,13,14,15]. Kabir et al. [1] emphasize carbon fibers’ prevalence in 3D-printed CFRCs, attributing their adoption to these mechanical advantages, particularly for lightweight structural components requiring high rigidity [9,10]. Hao et al. [8] demonstrate the mechanical superiority of 3D-printed carbon fiber-reinforced thermosetting composites over thermoplastic counterparts. Concurrently, Liu et al. [10] and Zhuang et al. [13] validate carbon fibers’ strength-enhancing capabilities through interfacial optimization with PA6 matrices and pre-impregnated filament development. Aerospace applications leverage carbon fibers’ weight reduction potential to enhance fuel efficiency [8,9], typically employing continuous fiber bundles or pre-impregnated filaments to maximize reinforcement [13,14]. Despite their advantages, high costs limit widespread adoption, driving research into hybrid systems [15].

Glass fibers provide cost-effective mechanical performance (2000–4000 MPa tensile strength, 70–90 GPa modulus) for construction, wind energy, and marine applications [16,17,18,19]. Their higher fracture strain enhances composite toughness compared to carbon fibers [16,17]. Popan et al. [16] experimentally verify 3D-printed glass fiber composites’ strength-to-weight advantages, while Yu et al. [17] achieve 48% flexural strength improvement through optimized PLA melt impregnation processes. The economic viability of glass fibers makes them attractive for mass-produced structural components [18,19], with reduced brittleness benefiting impact-resistant applications [19].

Aramid fibers (including Kevlar) exhibit exceptional strength, modulus, and thermal stability, finding applications in ballistic protection and aerospace [20,21]. Rijckaert et al. [20] substantiate their potential through mechanical characterization of 3D-printed aramid fiber composites.

Natural fibers (flax, hemp, and bamboo) gain attention for sustainability and biodegradability in eco-friendly and biomedical fields [22,23,24,25,26,27,28]. Despite lower tensile strengths (300–1000 MPa for flax), their low density (1.2–1.5 g/cm^3^ vs. 1.5–1.8 g/cm^3^ for carbon fibers) suits lightweight applications [23,24]. Recent advancements in 3D printing natural fibers show significant progress. Cai et al. [23] and Cheng et al. [24] have investigated continuous ramie fiber-reinforced biocomposites, demonstrating dynamic strength and shape memory effects that highlight their environmental applications. Long et al. [26] proved that optimized processing enables high-performance continuous flax/PLA composites with mechanical properties comparable to synthetic fiber composites in specific aspects, revealing natural fibers’ potential for demanding applications. Furthermore, 3D-printed continuous plant fiber composites exhibit unique recovery behaviors like shape memory and self-healing, which are particularly advantageous for applications requiring durability and resilience. Long et al. [28] further elucidated how printing parameters regulate the recovery mechanisms of flax fiber composites, providing critical insights for process optimization.

An emerging trend in research involves hybrid fiber systems, which combine different fiber types to optimize performance. For instance, Celik et al. [21] demonstrated that basalt/aramid hybrid composites achieve mechanical properties comparable to carbon fiber composites while offering cost advantages and enhanced impact toughness. This hybrid approach expands material options for 3D-printed CFRCs, particularly in applications requiring balanced performance and cost-effectiveness. Chen et al. [19] investigated carbon/glass fiber hybrid-reinforced PLA composites, revealing that glass fiber incorporation improves material toughness through synergistic effects.

Fiber surface treatment constitutes another critical consideration. Studies confirm that surface treatments—including coating and chemical modification—significantly enhance fiber–matrix adhesion, thereby improving composite performance [10,18]. For example, appropriate sizing treatments increase interfacial strength, optimizing load transfer efficiency [18]. Furthermore, the strategic combination of diverse fiber types and biomimetic designs provides application-specific adaptability [15,21,29].

### 2.2. Matrix Materials

The matrix plays a pivotal role in CFRCs by binding fibers and transferring loads, with its selection critically determining both composite performance and processing characteristics. Matrix materials fall into two categories: thermoplastics and thermosetting polymers, each possessing distinct advantages and limitations. Rational matrix selection not only governs mechanical properties but also dictates 3D printing feasibility and final application scopes.

#### 2.2.1. Properties of Matrix Materials at Room Temperature

Thermoplastics are particularly suited for 3D printing due to their processability and recyclability. As shown in Table 2, common thermoplastic matrices include polylactic acid (PLA), polyamide 6 (PA6), Acrylonitrile Butadiene Styrene (ABS) and polyetheretherketone (PEEK). PLA, valued for its biodegradability and low cost, finds extensive use in prototyping and biomedical applications [14,22,26]. However, its limited mechanical properties and low thermal stability restrict high-performance applications. In contrast, PA6 and PEEK are widely employed in demanding sectors like aerospace and automotive industries due to superior mechanical performance and thermal stability [10,11,13].

Recent advancements in PEEK-based CFRCs have yielded substantial progress. Studies demonstrate that optimized printing parameters and post-processing techniques (e.g., heat treatment) can significantly enhance PEEK composites’ mechanical properties [30]. Heat treatment improves crystallinity and reduces internal stresses, thereby increasing strength and stiffness. For instance, Wang et al. [30] achieved an 85% improvement in interlaminar shear strength through thermal processing. Low-melt polyaryletherketone (PAEK) has emerged as a potential PEEK alternative, exhibiting favorable tensile and thermal properties when combined with continuous carbon fibers [31]. Interlayer adhesion—a critical challenge in 3D-printed composites—has been addressed through optimized printing parameters and material formulations, enhancing printability and interfacial bonding in carbon/PEEK systems [32]. Concurrently, advancements in melt impregnation and fiber sizing have enabled high-performance PEEK filament production [33]. These technological breakthroughs collectively propel PEEK-based composites toward high-performance applications.

ABS has recently gained significant attention in natural fiber-reinforced composites. Due to its excellent processability and dimensional stability, ABS is widely used to prepare lightweight and eco-friendly natural fiber composites such as kenaf/ABS, oil palm/ABS, and cotton fiber/ABS. For example, Han et al. [34] investigated the mechanical properties of kenaf fiber-reinforced ABS composites with different fiber volume fractions and found that the incorporation of kenaf fibers reduced both tensile and flexural properties due to poor fiber–matrix interfacial adhesion, leading to delamination and porosity. Nevertheless, natural fiber-ABS composites still hold potential for fabrication via FDM technology and demonstrate promising prospects in the field of green composites.

Thermosetting plastics, such as epoxy and phenolic resins, are utilized in applications requiring long-term stability due to their enhanced temperature resistance and superior mechanical properties [8,35]. Although these resins are mature in traditional composite manufacturing processes like prepreg molding and resin transfer molding (RTM), their adoption in 3D printing remains limited. This limitation stems from the crosslinked networks formed during curing, which provide excellent stiffness and chemical resistance but complicate rapid curing control during printing—a critical requirement for ensuring uniform curing and final performance. Recent research efforts have developed in situ curing techniques and pre-impregnated fibers to streamline 3D printing of thermosetting composites. For example, Dong et al. [35] fabricated carbon fiber-reinforced phenolic composites using in situ curing technology, optimizing pre-curing temperatures to minimize defects. Despite these advancements, challenges persist in matching curing times with printing speeds and controlling shrinkage during curing processes [8,35].

#### 2.2.2. Mechanical Performance Under High-Temperature Conditions of Matrix Materials

Under elevated temperature conditions, polymer materials exhibit significant variations in mechanical performance. PLA, as a biodegradable polymer, demonstrates a Vicat softening temperature (VST) of approximately 59 °C, which restricts its applications in high-temperature environments. However, Kiatiporntipthak et al. [36] enhanced its thermal stability by blending with epoxy resin, increasing the VST to 64.6 °C. PA6 maintains superior mechanical properties at elevated temperatures, with research indicating its impact strength reaches peak values at 140 °C, reflecting excellent high-temperature toughness [37]. PEEK exhibits exceptional thermal stability and mechanical properties. Luo et al. [11] investigated the impregnation behavior of carbon fibers in PEEK matrices, reporting that PEEK stands out with continuous service temperatures up to 250 °C and exceptional chemical resistance, making it ideal for extreme environments. ABS shows a VST range of 90–105 °C, slightly higher than PLA but inferior to PA6 and PEEK, indicating relatively limited thermal stability. In contrast, epoxy and phenolic resins, as thermosetting polymers, form highly crosslinked networks after curing, endowing them with enhanced thermal resistance. The heat resistance of epoxy resins depends on curing agent selection and crosslink density, typically maintaining adequate mechanical performance within 150–200 °C. Phenolic resins, characterized by aromatic structures and carbonization tendencies, preserve stable mechanical properties even at 200–250 °C or beyond, making them suitable for applications demanding superior heat and fire resistance.

#### 2.2.3. Compatibility of Matrix Materials with 3D Printing Processes

When selecting matrix materials, researchers must prioritize compatibility with 3D printing processes. For thermoplastic matrices, melt viscosity and flow behavior must permit adequate fiber impregnation during printing [10,11,17]. For instance, PEEK’s high melting point (approximately 343 °C) necessitates high-temperature printing systems, whereas PLA’s lower melting temperature (around 180 °C) suits conventional equipment [11,22]. Thermosetting matrices require precise alignment between curing kinetics (crosslinking density development) and printing parameters to prevent under-cured or over-cured regions [35]. Additionally, matching the matrix’s coefficient of thermal expansion (CTE) with fibers minimizes thermal stresses and potential interlayer delamination [13]. These interdependent factors collectively determine a matrix’s applicability in specific scenarios.

### 2.3. Interfacial Treatment

**Figure 1:**
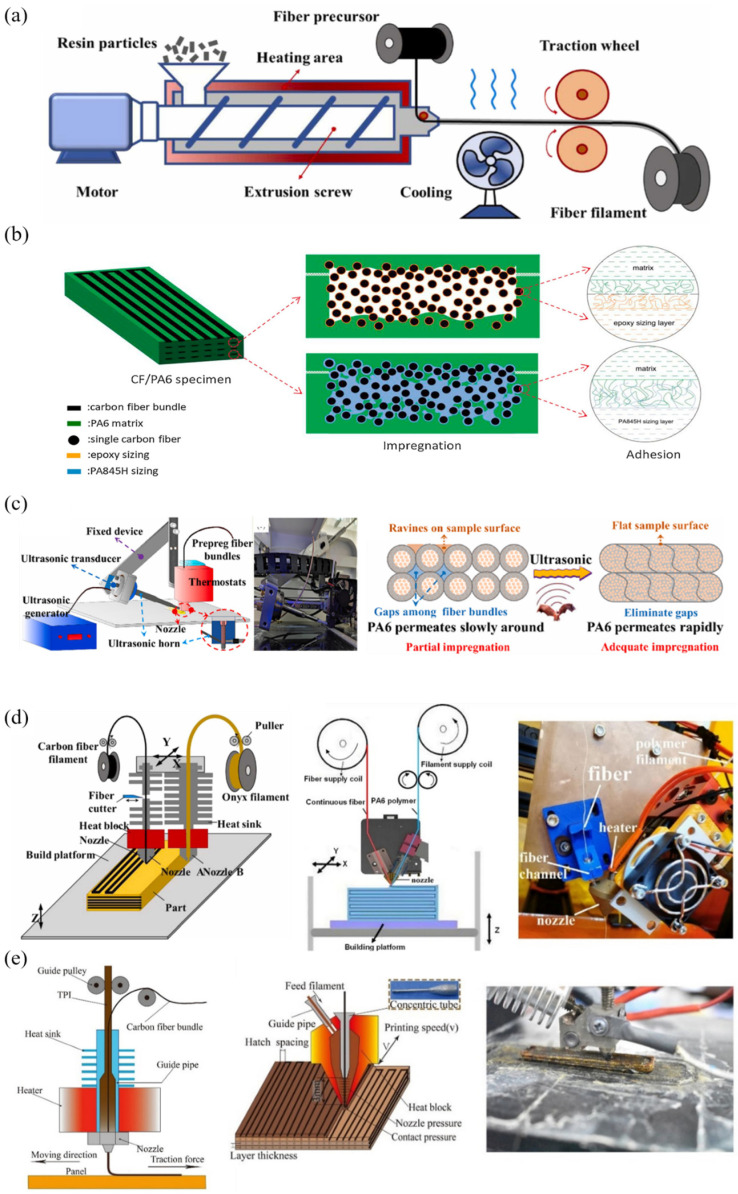
Interface treatment technologies (**a**) Preparation of continuous fiber prepreg filaments through resin impregnation [13]. (**b**) Enhancement mechanism of sizing treatment on CF/PA6 interfacial properties [10]. (**c**) Schematic diagram of ultrasonic-assisted device for improving CGF/PA6 interfacial performance during impregnation [18]. (**d**) Dual-nozzle system configuration [13,42]. (**e**) In situ impregnation apparatus [43].

Interface treatment constitutes another critical approach for enhancing 3D-printed composite performance. Surface coating or chemical modification of fibers can improve wettability and adhesion with the matrix, thereby boosting overall composite properties [10,18,38]. In this context, the process of preparing pre-impregnated fiber filaments by pre-wetting with resin, as shown in Figure 1a,b, has been developed. The development of pre-impregnated fibers, such as PA6-coated carbon fiber filaments, significantly enhances printing efficiency and mechanical performance [13,14]. While these techniques elevate material quality, they concurrently increase manufacturing complexity, necessitating cost–benefit analyses [15,39].

Fiber impregnation critically influences composite performance. Conventional 3D printing methods often struggle to achieve complete impregnation, resulting in voids or interfacial weaknesses. Therefore, recent studies have focused on optimizing the impregnation process, including the use of ultrasonic waves to enhance interfacial bonding performance during impregnation, as shown in Figure 1c. Additionally, the in situ needle-assisted melt impregnation [40] and dual-nozzle systems [41], as shown in Figure 1d,e, has been explored, which enhance fiber infiltration during printing. These advancements improve molten matrix penetration, strengthening mechanical properties and structural integrity. The dual-nozzle system exemplifies this progress by enabling simultaneous processing of dry fibers and matrix materials, optimizing impregnation quality [41] and achieving substantial tensile strength improvements.

Natural fiber composites require specialized processing optimization due to their hydrophilicity and thermal sensitivity. Research demonstrates that modified fused filament fabrication processes enhance printability and performance of continuous natural fiber-reinforced thermoplastics. For instance, proper drying protocols and surface treatments reduce moisture absorption while improving matrix adhesion [25]. These developments not only support natural fiber applications in 3D printing but also advance sustainable composites for biomedical devices and lightweight structures.

### 2.4. Challenges in Material Selection

Material selection for 3D-printed CFRCs involves multifaceted challenges that impact not only material performance but also processing feasibility and economic viability. Ensuring robust fiber–matrix adhesion stands as a primary challenge, as interfacial properties directly govern stress transfer and composite integrity [10,13,44]. Studies reveal that inadequate interfacial bonding may induce delamination and performance degradation [44,45]. To address this, researchers have developed surface modification techniques—including chemical treatments, plasma processing, and sizing agents—to enhance interfacial adhesion. For instance, surface modifications in shape-memory alloy wire composites substantially improve interfacial bond strength and mechanical performance. Gopalakrishnan et al. [38] systematically reviewed these surface engineering methods, establishing theoretical frameworks for fiber–matrix interface optimization.

Matrix compatibility with 3D printing processes presents another critical challenge. Thermoplastic matrices require precise control of melt viscosity and flow behavior to ensure proper fiber impregnation [17,18]. Excessive viscosity causes incomplete fiber wetting, while insufficient viscosity compromises dimensional accuracy. For thermosetting matrices, curing kinetics must synchronize with printing speeds and temperatures to prevent defects like uneven curing [35]. Temperature regulation during printing proves essential to mitigate thermal stresses and warping deformation [17,18]. Furthermore, fiber impregnation quality depends on optimized printing parameters (temperature, pressure), necessitating careful tuning to eliminate voids or dry spots [11].

Balancing cost and performance emerges as another pivotal challenge. While carbon fiber/PEEK matrix combinations deliver exceptional performance, their high costs may restrict widespread market adoption [9,15]. Conversely, glass fiber/PLA systems offer economic advantages but often lack the mechanical robustness required for high-load applications [16,17]. This dilemma drives research into hybrid material systems that combine diverse fibers or matrices to optimize cost-performance ratios [21,46]. For example, Hou et al. [46] investigated coaxial continuous hybrid fiber composites, demonstrating significant impact strength improvements through hybrid effects. Beyond conventional polymer matrices, researchers are exploring unconventional alternatives like low-melting-point alloys. 3D-printed continuous fiber-reinforced metal matrix composites exhibit unique properties such as electrical conductivity and ductility [47], though fiber compatibility and process adaptability require further investigation.

The sustainability–performance trade-off presents additional complexities. Natural fibers and bio-based matrices like PLA, despite their environmental benefits, often underperform traditional materials in mechanical durability [23,24,25,26,28]. Touchard et al. [27] analyzed continuous cellulose fiber composites’ microstructures, revealing how natural fibers’ hygroscopicity compromises long-term performance. Recycling and reusability remain unresolved challenges, though recent breakthroughs in supercooled polymer melt processing enable fully recyclable continuous fiber self-reinforced composites that permit matrix/fiber reuse [48]. However, whether these sustainable materials can meet high-load demands awaits empirical validation.

This section has examined critical material selection aspects for 3D-printed CFRCs. Carbon, glass, aramid, and natural fibers are widely adopted for their unique mechanical/cost profiles, while matrices must ensure both process compatibility and interfacial adhesion. Challenges in impregnation optimization and cost-performance balancing underscore the necessity for technological innovation and strategic trade-offs in developing high-performance composites.

## 3. Manufacturing Technologies and Processes

Additive manufacturing technologies can be primarily categorized into three types: solid-based (e.g., FDM filament extrusion), liquid-based (e.g., SLA/DLP photopolymerization), and powder-based (e.g., SLS/SLM). Currently, Fused Deposition Modeling (FDM, also named Fused Filament Fabrication (FFF)) remains the most mature solution for continuous fiber 3D printing. Emerging research has begun exploring continuous fiber integration in photopolymerization processes (e.g., UV-curable prepregs or post-printing fiber insertion), while powder-based methods remain unsuitable due to their inherent inability to maintain fiber continuity and alignment. FDM currently dominate industrial applications due to their accessibility and material versatility [10,49,50,51]. The growing demand for high-performance composites has driven FDM’s expanded adoption in aerospace, automotive, and medical sectors [48,51]. Emerging techniques like stereolithography (SLA) and ultraviolet (UV)-cured resin methods are being explored, offering novel manufacturing possibilities for continuous fiber composites [51,52]. Those technologies are summarized in Table 3.

### 3.1. FDM/FFF Techology

**Figure 2:**
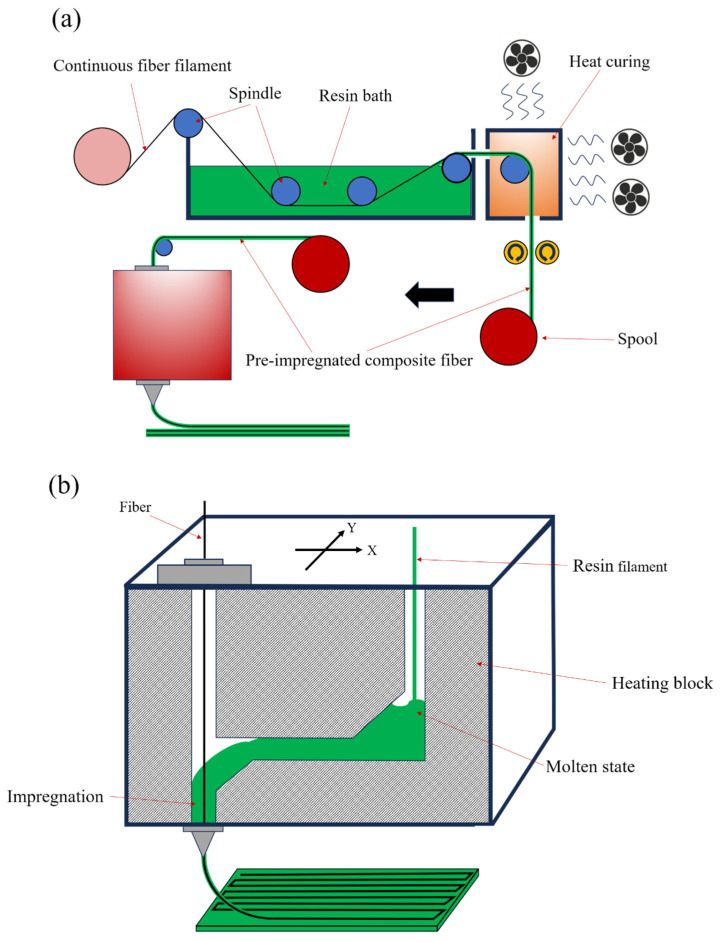
In situ impregnation method versus pre-impregnated filament method. (**a**) Pre-impregnated filament method. (**b**) In situ impregnation method.

The FDM/FFF process, a material extrusion-based additive manufacturing method, constructs components by melting and extruding thermoplastic filaments through nozzles in layerwise deposition. Its cost-effectiveness and material diversity have garnered substantial attention [51,53]. For continuous fiber composites, FDM primarily employs two strategies, as shown in Figure 2: pre-impregnated filament processing and in situ impregnation, each presenting distinct advantages and challenges [54,55,56].

The pre-impregnated filament approach utilizes continuous fibers pre-coated with matrix materials, forming composite filaments compatible with standard FDM printers [11,54]. During printing, heated filaments are extruded through nozzles, depositing reinforced materials along programmed paths [55,57]. This method substantially enhances tensile strength and stiffness—Liu et al. demonstrated several-fold mechanical improvements in continuous carbon fiber/PA6 composites compared to pure polymers [10]. Akhoundi et al. [58] further validated that optimized parameters achieve 50% fiber volume fractions, yielding tensile strength and modulus of 478 MPa and 29.4 GPa, respectively. However, fiber misalignment and breakage may occur in small-diameter nozzles, particularly under excessive melting temperatures [55,59]. Qiao et al. utilized ultrasonic waves to improve the impregnation effect of the matrix and fibers [60]. Cheng et al. addressed this by developing high-performance pre-impregnated tows through optimized fiber distribution, enhancing printing efficiency and part quality [61]. Zhang et al. reduced fiber defects via advanced deposition techniques [62], while precise path planning enables complex architectures like fiber-reinforced lattices and honeycombs [63,64].

The in situ impregnation method differs fundamentally from pre-impregnated approaches by feeding continuous fibers through independent channels within the print head while depositing matrix materials around the fibers during extrusion [57,65]. This technique avoids fiber compression through narrow nozzles, thereby preserving fiber continuity and alignment [66,67]. In situ impregnation proves particularly advantageous for applications requiring precise fiber placement, such as lightweight aerospace components [12,68]. He et al. demonstrated that 3D-printed continuous fiber-reinforced thermosetting composites via in situ impregnation achieve mechanical strength comparable to direct-molded counterparts [12]. Furthermore, dynamic capillary-driven additive manufacturing leverages capillary forces to control polymer viscosity and curing processes, enabling rapid impregnation and in situ solidification—especially suitable for complex geometries [69,70]. Zhang et al. found that this method effectively reduces void content and enhances interfacial properties when fabricating intricate structures [70]. Recent advancements extend FDM technology to thermosetting composites. Hao et al. [8] developed continuous carbon fiber-reinforced thermosetting resin composites with mechanical properties surpassing traditional thermoplastic matrix systems. Liu et al. [71] further improved printing accuracy by 90% through optimized corner fiber deposition algorithms, establishing critical foundations for high-performance applications.

Variants of FDM technology have significantly advanced CFRP applications. Ultrasonic-assisted printing enhances interfacial bonding by improving matrix wetting and fiber impregnation [66,72]. Qiao et al. achieved 34% and 29% improvements in tensile and flexural strength, respectively, through ultrasonic treatment [60]. Microwave-assisted printing accelerates manufacturing speeds via rapid heating and matrix curing [67,73]. Li et al. demonstrated printing velocities multiple times faster than conventional methods while maintaining mechanical integrity using microwave heating [67]. To enhance interlayer adhesion, sinusoidal path extrusion 3D printing modifies deposition amplitude and frequency, substantially improving performance in continuous carbon fiber-reinforced polylactic acid (PLA) composites. Experimental results reveal 95.4%, 57.3%, and 165% increases in tensile strength, modulus, and fracture energy absorption, respectively [74,75]. Robotic arm-based continuous fiber 3D printing has achieved breakthroughs through multi-axis deposition on complex surfaces, expanding FDM’s applicability [76,77]. Abdullah et al. successfully fabricated geometrically intricate thermosetting composites using robotic systems, enabling large-scale production [76]. Akhoundi et al. attained 0.1 mm maximum deviation in continuous glass fiber deposition on curved surfaces through G-code optimization [77].

### 3.2. Photopolymerization Methods

**Figure 3:**
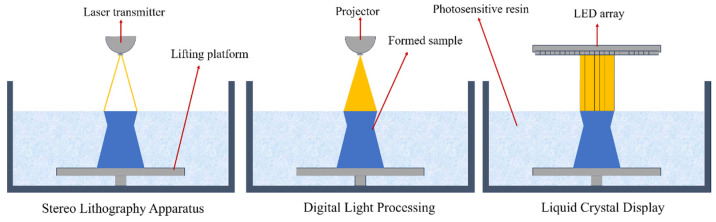
Schematic diagram of photopolymerization-based 3D printing technology for composites.

Photopolymerization techniques, including stereolithography (SLA) and digital light processing (DLP), fabricate components by layerwise curing of liquid photopolymer resins under ultraviolet (UV) light [49,51], as shown in Figure 3. Compared to FDM, integrating continuous fibers into photopolymerization processes presents greater complexity due to challenges in precise fiber alignment caused by the liquid-state resin properties [78].

A conventional approach involves manually or mechanically placing fibers on the build platform before each layer’s curing [78,79]. However, achieving continuous fiber reinforcement across multiple layers requires precise interlayer fiber alignment, presenting significant technical challenges [80]. Alternative strategies employ short-fiber-reinforced resin slurries, but these only provide discontinuous reinforcement, failing to exploit continuous fibers’ full potential [73,80]. To overcome these limitations, resin vat fiber feeding systems integrated with robotic positioning have been developed, substantially improving placement accuracy [79,81]. Quan et al. achieved micron-level fiber positioning in liquid resins using robotic assistance [79], while Rahman et al. enhanced mechanical performance through optimized fiber placement protocols [81].

Recent innovations have expanded photopolymerization’s capabilities for continuous fiber composites. Two-stage ultraviolet (UV) curing strategies first immobilize fibers via partial curing before completing polymerization, enabling efficient reinforcement [73,82]. Li et al. demonstrated this method’s effectiveness through continuous carbon fiber composites with improved alignment precision and mechanical properties [73]. Jiang et al. further validated its potential for high-fiber-content composites, achieving strengths comparable to molded counterparts [82]. Nevertheless, the inherent complexity of fiber manipulation in liquid media continues to constrain photopolymerization’s adoption for continuous fiber reinforcement [49].

**Table 3:** Technical classification of 3D printing methods for CFRPs.

Technology Classification	Core Method	Characteristics	References
**FDM Technology**			
**Pre-impregnated filament method**	Uses pre-impregnated fiber filaments	High tensile strength and stiffness, but prone to fiber breakage/misalignment	[10,11,54,55,56,57,58,59,60,61,62,63,64]
**In situ ** **impregnation method**	Independent fiber feeding with simultaneous matrix deposition	High-precision fiber alignment, but complex process control	[12,57,65,66,67,68,69,70,71]
**FDM Technology Variants**			
**Ultrasonic-assisted printing**	Ultrasonic-enhanced fiber wetting	Improved interfacial bonding, but requires complex equipment	[66,72]
**Microwave-assisted printing**	Microwave rapid heating and curing	Faster printing speed, requires specialized equipment	[67,73]
**Sinusoidal path ** **extrusion**	Path optimization for interlayer bonding	Enhanced interlayer performance, but complex algorithms required	[74,75]
**Robotic arm ** **technology**	Multi-axis deposition for complex surfaces	High precision for complex structures, but costly	[76,77]
**Photopolymerization Methods**			
**SLA/DLP**	UV curing of liquid resin	High precision, but challenging for continuous fiber alignment	[49,51,78,79,80,81]
**Two-stage UV ** **curing**	Stepwise curing for fiber fixation	High fiber alignment precision, but multiple process steps	[73,82]
**Resin bath fiber feeding**	Robotic fiber positioning in liquid resin	Excellent mechanical properties, but difficult to process	[79,81]

### 3.3. Critical Process Parameters

The successful application of 3D-printed continuous fiber composites hinges on precise control of key process parameters that directly determine printing quality and composite performance [35,54], as shown in Table 4.

Printing Speed: Printing speed governs fiber placement accuracy and matrix distribution. Excessive speeds increase void content, compromising part strength [68,83]. For continuous glass fiber composites, reduced speeds optimize fiber distribution and impregnation quality [17,84]. Yu et al. established 300 mm/min as the optimal speed for uniform fiber distribution through heating zone length analysis [17], while Liu et al. enhanced deposition precision at elevated speeds via path-driven designs [84].

Nozzle Temperature: Temperature optimization balances matrix melting and fiber integrity. Carbon fibers tolerate high temperatures, but low-melting polymers like polylactic acid (PLA) risk degradation at excessive heat [85,86]. Precise thermal control achieves optimal impregnation without fiber damage [87,88]. Chen et al. minimized fiber misalignment through adaptive temperature optimization [87], and Quan et al. defined temperature ranges for various geometries using fiber bundle deposition models [88].

Fiber Feed Rate: Synchronizing fiber feed and matrix extrusion rates ensures consistent fiber volume fractions and reinforcement uniformity [89,90]. Rate mismatches cause fiber accumulation or matrix deficiency, degrading performance [91]. Liu et al. demonstrated minimal void content through synchronized control experiments [91].

Path Planning: Fiber orientation dictated by path planning critically influences composite anisotropy. Load-adaptive path planning enhances mechanical properties by aligning fibers with stress directions—increasing fiber density in tensile regions [94,95]. Wang et al. achieved 40.9% tensile strength improvement via geometry-driven path optimization [96], while Wein et al. enhanced flexibility through multi-layer fiber arrangements [97].

Layer Thickness: These parameters affect resolution and strength. Thicker layers improve fiber alignment but reduce surface finish, whereas thinner layers suit high-precision applications [75,98]. Ding et al. [90] identified 30–40% fiber diameter as the optimal layer thickness for balancing flexural strength and surface quality.

In summary, fused deposition modeling (FDM/FFF) remains dominant for 3D-printed continuous fiber composites, while photopolymerization faces persistent challenges. Process success fundamentally depends on precise control of printing speed, nozzle temperature, fiber feed rate, and other critical parameters.

## 4. Mechanical Properties

The mechanical performance of 3D-printed CFRPs serves as the foundational criterion for their widespread application in aerospace, automotive manufacturing, and structural engineering. Compared to traditional manufacturing, 3D printing’s layerwise deposition and controlled fiber paths endow materials with unique tensile strength, compressive strength, flexural modulus, impact toughness, and interlaminar bonding strength. These properties directly determine CFRCs’ reliability and applicability under complex loading conditions. This section comprehensively analyzes key factors influencing mechanical performance—including fiber type, fiber volume fraction, fiber orientation, printing parameters, and post-processing (as shown in Table 5)—while evaluating optimization strategies and practical outcomes based on recent research to provide theoretical and practical guidance for enhancing CFRCs’ mechanical capabilities.

Fiber type is one of the fundamental factors determining the mechanical properties of CFRCs. Different fibers (e.g., carbon, glass, and aramid fibers) exhibit significant variations in tensile strength, compressive strength, flexural modulus, and impact toughness due to their inherent mechanical characteristics. Extensive studies have shown that carbon fiber-reinforced composites (CFRCs) generally outperform glass or natural fiber-reinforced composites under tensile loading. Mohammadizadeh et al. [99] evaluated the tensile properties and failure mechanisms of nylon composites reinforced with carbon, glass, and Kevlar fibers. They found that increasing fiber content enhanced tensile performance, with carbon fibers providing superior reinforcement compared to glass and Kevlar fibers. Silva et al. [100] conducted three-point bending tests on carbon fiber (CF) and glass fiber (GF)-reinforced sandwich structures, revealing that CF sandwich panels exhibited 25% higher flexural strength and 70% higher flexural modulus than GF panels. When the fiber volume fraction increased from 13% to 26%, the bending strength and modulus improved by 30%. However, challenges such as fiber–matrix voids and fiber curvature during 3D printing may induce fiber damage, leading to conflicting conclusions under different experimental conditions. Contrary to Silva et al. [100], Alarifi et al. [101] evaluated the flexural properties of nylon composites reinforced with carbon and glass fibers, showing that the GF-reinforced nylon composite (nylon/GF) exhibited higher flexural strength and stiffness than the CF-reinforced counterpart (nylon/CF) at 0° fiber orientation, suggesting better-bending resistance. Ning et al. [102] compared the impact performance of Kevlar- and glass fiber-reinforced samples under identical printing parameters, finding that glass fiber samples absorbed 77% more energy than Kevlar-reinforced ones. This contrasts with the conventional understanding that Kevlar composites typically exhibit superior impact resistance. Mousapour et al. [103] fabricated continuous carbon fiber (CCF)-reinforced bronze-matrix composites, observing that while CCF improved tensile strength and Young’s modulus, excessive voids caused by CCF reduced tensile strength by 23%.

Fiber volume fraction, as another critical parameter, exhibits a nonlinear relationship with mechanical properties and may trigger distinct failure mechanisms at different levels. Gracego et al. [104] reported that increasing fiber volume fraction shifted the failure mode of CFRCs from fiber/matrix fracture to fiber–matrix debonding, requiring lower printing speed at higher fractions. Generally, higher fiber volume fractions enhance tensile and compressive strength. Wang et al. [105] investigated the effects of fiber volume fraction on the tensile and flexural properties of continuous glass fiber-reinforced PLA composites. At 5.21% fiber volume fraction, the tensile strength increased by 400% compared to pure PLA. Araya-Calvo’s team [54] evaluated the influence of reinforcement methods and fiber volume fraction on the compressive and flexural properties of continuous carbon fiber-reinforced polyamide 6 (PA6). A fiber volume fraction of 48.93% improved the bending strength by 61.27% and 176.8% compared to fractions of 32.19% and 17.18%, respectively. Compressive strength also increased proportionally with fiber volume fraction from 8.18% to 24.44%. However, some studies indicate that mechanical properties enhancement with increasing fiber volume fraction has an optimal range. For example, Zhuang et al. [106] observed a parabolic relationship between fiber volume fraction and shear/tensile strength, where excessive fiber content increased porosity and weakened interlayer bonding, limiting further improvements. Other studies optimized fiber distribution for specific loading conditions. Fedulov et al. [107] proposed a topology optimization framework for fiber density and orientation in continuous fiber 3D printing. Hou et al. [108] designed localized fiber volume fractions based on stress distribution, achieving 11% and 13% increases in flexural strength and modulus compared to homogeneous structures.

Printing parameters (e.g., nozzle temperature, speed, layer thickness, and infill density) indirectly but significantly regulate CFRCs’ mechanical properties by affecting interlayer adhesion and fiber quality. Dou et al. [85] and Wang et al. [105] studied the influence of temperature, speed, layer height, and fiber volume fraction on the tensile and flexural properties of continuous glass fiber-reinforced PLA composites. Smaller layer heights and extrusion widths increased relative fiber content, enhancing tensile strength and stiffness. Rahman et al. [109] investigated the influence of process parameters on the mechanical properties of 3D-printed continuous fiber-reinforced thermoset composites. They tested tensile and flexural properties under various nozzle diameters (0.8 mm, 1.0 mm, and 1.2 mm) and printing spacings (0.75–1.1 mm). The results showed tensile strengths ranging from 148 to 232 MPa and Young’s moduli between 21.1 and 31.2 GPa. Flexural strength and modulus varied from 220.2 to 345.7 MPa and 10.2 to 16.5 GPa, respectively. The optimal mechanical properties (tensile strength of 232 MPa and flexural strength of 345.7 MPa) were achieved with the smallest nozzle diameter (0.8 mm) and minimal printing spacing (0.75 mm) combination. Pervaiz’s team [110] identified an optimal parameter set to maximize the mechanical strength and energy absorption capacity of 3D-printed components. Their study demonstrated that carbon fiber-reinforced nylon specimens printed under the following conditions exhibited the best mechanical performance and energy absorption rate: 80% infill density, 0° fiber orientation, 12–13 carbon fiber layers, and a strain rate of 10 mm/min. The research further quantified parameter contributions to mechanical performance: fiber orientation accounted for 56.13% of variance, followed by infill density (16.25%) and fiber layer position (10.12%). Duan et al. [111] developed a system for 3D printing continuous fiber-reinforced cementitious composites (CFRCCs) and analyzed the effects of speed, extrusion rate, layer thickness, and nozzle diameter on flexural strength. Ghnatios et al. [112] found that increasing platform and nozzle temperature reduced edge deformation and residual thermal stress. Ferreira et al. [113] reported that aged specimens exhibited lower elastic modulus, reduced visible damage, and diminished energy absorption. Kuncius et al. [114] and Wu et al. [115] demonstrated that reducing layer height from 0.4 mm to 0.3 mm improved shear strength by 40%, while flexural properties peaked at 0.7–0.9 mm. To address layer thickness effects, Wang et al. [116] proposed a variable-thickness printing method for honeycomb cores, achieving compressive strength and specific energy absorption of 38.1 MPa and 25.4 kJ/kg, respectively. Beyond FDM, Ipekci et al. [117] optimized UV intensity, nozzle diameter, and speed for stereolithography, producing parts with tensile and flexural strengths of 125 MPa and 450 MPa.

Fiber orientation critically governs CFRCs’ mechanical performance, with significant variations under identical loads. Naik et al. [118] found that unidirectional 0°-oriented samples exhibited 439.47% higher impact strength than other orientations. Man et al. [119] confirmed that fiber orientation dominates the wear resistance of 3D-printed CCF/PA6 composites. Kim’s team [120] showed that the precise alignment of continuous carbon fibers in sandwich cores enhanced specific flexural strength, stiffness, and energy absorption. While uniform fiber distribution improves dimensional stability [121], complex geometries (e.g., notched or irregular structures) demand customized fiber paths to mitigate stress concentrations. Zhang et al. [122] proposed a method to simultaneously optimize topology, orientation, and density distribution, effectively dispersing stress and reducing failure probability. Yang et al. [123] improved stiffness and stiffness-to-weight ratios by 30.0% and 26.3% through fiber path optimization. Load-aligned fibers generally enhance strength and stiffness, as shown by Li et al. [124]. Zhang et al. [125] increased open-hole plate strength by 166% using stress trajectory-aligned fibers, redistributing stress concentrations and reducing deformation. Zhang et al. [126] achieved 305% and 256% improvements in flexural strength and stiffness via topology-optimized fiber paths. However, Guan et al. [127] developed a “core interleaved alignment” strategy, increasing honeycomb compressive strength and modulus by 96% and 67.5%, respectively. Cheng et al. [128] designed three cell-crossing paths for bio-composite honeycombs, optimizing compression, bending, and tensile performance. Chen et al. [129] established a predictive model for damage behavior in CFRCs with complex fiber paths.

Interfacial issues remain a critical challenge in 3D-printed CFRCs due to layer-by-layer fabrication. Dang et al. [130] examined fracture behaviors of specimens with carbon/carbon, Kevlar/Kevlar, and carbon/Kevlar hybrid interfaces. Their findings revealed that carbon/carbon interface specimens exhibited unstable crack propagation, while specimens with Kevlar/Kevlar and carbon/Kevlar hybrid interfaces showed significantly improved Mode I interlaminar fracture toughness due to fiber bridging zones formed by Kevlar fibers. This suggests Kevlar fibers could serve as transition materials to enhance interfacial properties in composites. Kong et al. [131] and Yadav et al. [132] investigated interfacial responses under mixed stresses, revealing significant variations in material and fiber angles. Li et al. [133] found that central fiber stacking under 0° raster angles enhanced tensile strength via better interfacial bonding, while 45° angles favored edge stacking. Zhao’s team [134] modeled interfacial debonding in realistic 3D-printed composites. Lee et al. [135] reduced void defects by filling carbon fiber bends with Onyx, improving interfacial quality. Zhang et al. [136] enhanced CCF/TPU interfacial bonding via wet-twisting, increasing tensile strength and modulus by 62.18% and 87.16%.

To further enhance CFRCs, researchers have explored fiber pretreatment, structural optimization, and hybrid strategies. Ye et al. [137] investigated the response of honeycomb structures under low-velocity impact, revealing that continuous carbon fibers effectively suppressed the initiation and propagation of impact damage, resulting in superior impact resistance of CCFR (continuous carbon fiber reinforced) honeycomb structures. Dou et al. [138] fabricated lightweight honeycomb structures including CCFR honeycomb, aluminum alloy honeycomb, and pure PLA honeycomb, and conducted compression experiments. Under longitudinal loading, the specific energy absorption of CCFR honeycomb was measured to be 186.58% and 596.84% higher than that of pure PLA and aluminum alloy honeycombs, respectively. Vattathurvalappil et al. [139] evaluated the mechanical properties of specimens with mechanically drilled holes versus 3D-printed holes. Their study identified delamination caused by hole drilling as the primary factor for tensile strength reduction. The 3D-printed holes significantly mitigated this delamination phenomenon. Furthermore, increasing the stiffness around the hole area was found to effectively alleviate stress concentration. Fiber pretreatment (e.g., coatings or chemical modifications) enhances fiber–matrix adhesion. Jung et al. [140] optimized basalt fiber surface treatment with 3 wt% solution coating, achieving 175 MPa tensile strength. Islam et al. [141] demonstrated that improved resin impregnation reduced porosity and enhanced tensile properties. Li et al. [142] and Wang et al. [143] incorporated carbon nanotubes and graphene oxide to minimize voids and strengthen interfaces. Zhang et al. [144] reduced porosity from 2.69% to 0.06% via epoxy infusion, boosting CFR-PA6 tensile strength and stiffness by 22.1% and 29.3%. Rahman et al. [145] addressed UV shielding in photocurable resins using thermal initiators. Structural innovations include Toth et al. [146] and Nikiema et al. [147], who found that concentrated fiber/matrix stacking outperformed alternating layers. Giarmas et al. [148] enhanced honeycomb flexural strength by distributing glass fibers at the top/bottom instead of the core. Saniei et al. [149] reported that auxetic structures improved impact resistance but reduced tensile strength.

Hybrid fibers and advanced processes have also been explored. Wang et al. [150] optimized hybrid carbon/Kevlar fiber stacking for indentation resistance. Wang et al. [151] observed enhanced energy absorption in PA-based hybrid composites under quasi-static indentation. Ding et al. [152] traded 10% tensile strength for 230% impact strength by hybridizing carbon with 25% glass fibers. Liu et al. [153] achieved 15.3% and 92.4% tensile strength improvements in hybrid Kevlar/carbon composites. Ju et al. [154] demonstrated exceptional energy absorption in hybrid honeycombs. Liu et al. [155] highlighted deviations in traditional aging models for 3D-printed hybrid composites. Advanced techniques include Fu et al. [156], who developed an alternating deposition method for impact-resistant orthogonal fabric composites, and Rudykh’s team [157], who created shape-memory honeycombs with 87% recovery. Fu et al. [158] established multiscale models to predict CFR-TPC performance, integrating resin permeation, thermal effects, and curing dynamics.

In summary, CFRCs’ mechanical properties result from multifactorial interactions. Strategic selection of fiber types, optimization of volume fractions/orientations, parameter tuning, and post-processing/structural design collectively enhance tensile/compressive strength, flexural modulus, impact toughness, and interlaminar bonding.

## 5. Functionality

Beyond mechanical performance, 3D-printed CFRPs attract significant interest in their functional properties, including electrical conductivity, thermal management, self-sensing capabilities, and shape memory behavior.

### 5.1. Electrical Properties

**Figure 4:**
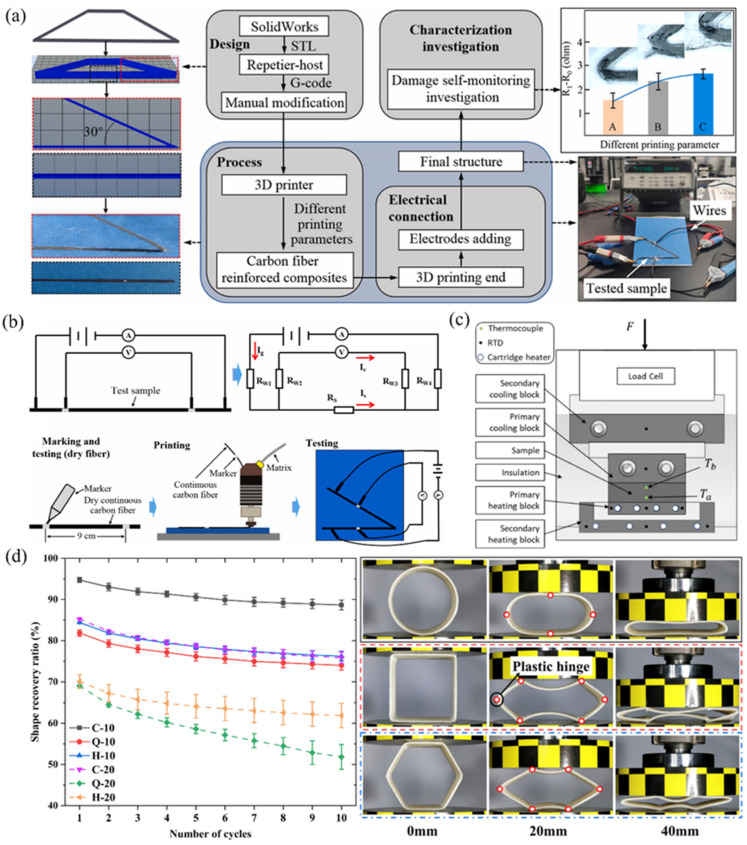
Functional characteristics of 3D-printed CFRPs (**a**) Self-monitoring of continuous fiber damage [160]. (**b**) Fiber damage monitoring through resistance variation [160]. (**c**) Thermal conductivity measurement methodology [162]. (**d**) Shape memory recovery functionality in continuous ramie fiber-reinforced composite structures [166].

Continuous carbon fibers are particularly notable for their high electrical conductivity, enabling the creation of 3D-printed composites with tailored electrical behaviors. Multiple studies have investigated these materials’ electrical characteristics. For instance, Galos et al. [159] analyzed the electrical conductivity of FDM-printed continuous carbon fiber composites, examining how printing parameters and fiber orientation influence conductivity. The study revealed that fiber breakage reduces conductivity, while increased fiber waviness enhances current flow in transverse and thickness directions. As shown in Figure 4a,b, Cheng et al. [160] explored damage monitoring via electrical resistance changes during the manufacturing of 3D-printed carbon fiber composites, highlighting their potential for self-sensing applications—such as defect detection through resistance variations.

Research has further extended to self-sensing under mechanical loads. Ye et al. [161] investigated the electrical self-sensing performance of 3D-printed continuous carbon fiber-reinforced PLA/TPU honeycomb structures during cyclic compression. Results showed reversible resistance increases proportional to compressive strain (attributed to cross-sectional area reduction and fiber damage), enabling strain monitoring, while irreversible resistance spikes indicated damage. Additionally, resistance exhibits linear temperature dependence, facilitating thermal monitoring. These multifunctional traits make such composites ideal for structures requiring real-time health assessment. Despite progress, challenges persist in ensuring stable electrical performance across printing conditions and maintaining fiber integrity. Future work should optimize printing parameters to minimize fiber damage and develop novel matrix materials to advance electrical functionality.

### 5.2. Thermal Properties

The thermal conductivity of 3D-printed CFRPs represents another critical functional attribute, particularly for heat dissipation and thermal management applications. Olcun et al. [162] investigated the thermal conductivity of 3D-printed continuous pitch carbon fiber composites as shown in Figure 4c. Renowned for their high thermal conductivity, pitch carbon fibers’ effective thermal conductivity was shown to depend significantly on fiber volume fraction and printing parameters. In a subsequent study, Olcun et al. [163] utilized a 6-axis robotic arm to print pitch carbon fiber composites, achieving 82% of the fiber’s intrinsic thermal conductivity (approximately 1000 W/m·K) by optimizing extrusion angles to minimize fiber breakage. This advancement demonstrates the potential for complex geometries in thermal management systems, such as electronics cooling. However, fiber damage during printing remains a limiting factor, necessitating precise alignment optimization.

Research on thermal performance also encompasses temperature effects on mechanical behavior. Talha et al. [164] conducted thermomechanical analyses of 3D-printed continuous glass fiber-reinforced Onyx thermoplastic composites. Onyx, a carbon-filled nylon, combined with glass fibers (lower thermal conductivity than carbon fibers), exhibited mechanical degradation at elevated temperatures but improved deformation resistance under thermal loading. Despite progress, long-term thermal stability and orientation-dependent thermal performance require further investigation. Additionally, practical integration demands understanding material behavior under real-world conditions like thermal cycling and environmental exposure.

### 5.3. Intelligent Functionality: Self-Sensing, Self-Monitoring, and Shape Memory/Recovery Behaviors

Self-sensing and self-monitoring capabilities significantly enhance composite structures’ functionality and safety. Study [165] proposed a dual-material 3D printing process to create self-monitoring continuous carbon fiber-reinforced thermoplastic composites, potentially involving integrated sensors or leveraging carbon fibers’ intrinsic properties for structural health monitoring (specific methodology unspecified in the cited work). Similarly, Cheng et al. [160] demonstrated damage detection during manufacturing through electrical resistance changes, exemplifying self-sensing applications.

Shape memory and recovery behaviors constitute another research frontier. As shown in Figure 4d, Lin et al. [166] investigated shape recovery in 3D-printed continuous ramie fiber-reinforced thin-walled biocomposite structures, achieving 89% shape recovery rates through optimized printing parameters and circular geometric designs, alongside stable energy absorption during cyclic loading. The unique properties of plant fibers like ramie—potentially linked to their natural hierarchical structures or processing methods—underpin these recovery behaviors.

Research further extends to environmentally responsive composites. De Kergariou et al. [167] designed 3D/4D-printed continuous fiber composites using evolutionary algorithms and voxel-based finite element methods for hygromorphic applications. By optimizing fiber orientation within individual voxels, the study achieved humidity-triggered shape memory effects that mimic natural hygromorphs, highlighting 3D printing’s potential in creating adaptive environmental-response composites.

In summary, the functional properties of 3D-printed CFRPs significantly expand their application potential. From electrical conductivity and thermal management to self-sensing capabilities and shape memory behaviors, these materials demonstrate substantial promise for advanced engineering applications.

## 6. Application Fields

3D-printed CFRPs demonstrate broad application potential across aerospace, automotive, and biomedical industries due to their exceptional performance characteristics. These materials not only exhibit high strength-to-weight ratios but also enable customized designs for specific functional requirements. This section comprehensively examines recent advancements in these domains, supplemented with additional relevant citations to fully illustrate current progress and future prospects.

### 6.1. Aerospace Applications

**Figure 5:**
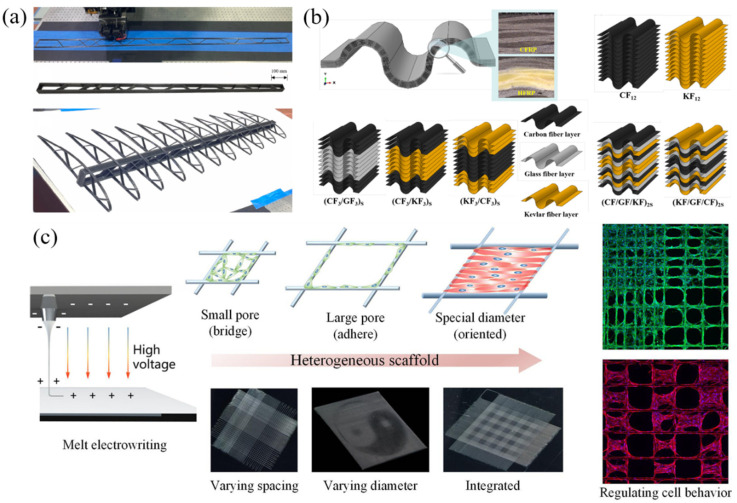
Applications of 3D-printed CFRPs: (**a**) 3D-printed continuous carbon fiber-reinforced composite wing [168]. (**b**) 3D-printed hybrid fiber-reinforced corrugated crash-resistant structure [172]. (**c**) 3D-printed scaffold structure for cell growth induction [177].

In the aerospace sector, 3D-printed CFRCs are highly valued for their superior strength-to-weight ratios, which reduce fuel consumption while enhancing overall performance. For instance, study [83] developed a specialized 3D printing system for aerospace structural components, particularly wing spars, demonstrating how precise fiber distribution optimizes structural efficiency. Furthermore, research [168] proposed topology-optimized spar designs integrated with continuous carbon fiber reinforcement, as shown in Figure 5a, achieving lightweight yet robust configurations that provide innovative solutions for aerospace parts. Impact resistance and energy absorption are critical for aviation safety. Study [169] investigated bio-inspired sandwich panels fabricated via monolithic 3D printing of continuous carbon fiber-reinforced polyamide, revealing exceptional energy absorption capabilities suitable for airframe and wing applications.

The scope of aerospace applications has been further expanded through advanced research. For instance, bio-inspired continuous carbon fiber-reinforced resin composites were developed [170], where strength and toughness were enhanced through biomimetic structural designs for high-durability aerospace components. The potential of bio-inspired designs was demonstrated [171], where continuous carbon fiber-reinforced polylactic acid (PLA) composites mimicking owl feather barbule structures were 3D-printed, achieving superior lightweight and impact-resistant properties. Additionally, recoverable honeycomb composites capable of restoring their original shape post-deformation were investigated [128], suggesting applications in adaptive or self-repairing aerospace structures. Similarly, corrugated-core sandwich structures in continuous carbon fiber-reinforced thermoplastic composites were explored [111], with stiffness and load-bearing capacity significantly improved for aerospace support components.

Nevertheless, challenges persist in aerospace applications, including long-term material stability under extreme environments and consistency in fiber distribution during manufacturing. Further optimization of printing processes is essential to meet stringent aviation standards.

### 6.2. Applications in Automotive Industry

In the automotive industry, lightweight materials are crucial for improving fuel efficiency and reducing emissions. 3D-printed CFRPs have garnered significant attention due to their design flexibility and exceptional crash resistance. For example, multi-fiber hybrid composite corrugated structures as shown in Figure 5b were investigated [172], combining carbon, glass, and Kevlar fibers, which revealed significant crash resistance improvements through synergistic effects, making them ideal for automotive safety components like energy-absorbing tubes. Research [173] further analyzed the performance and failure modes of continuous fiber-reinforced energy-absorbing tubes fabricated via cylindrical layered 3D printing, providing theoretical foundations for designing efficient crash buffers.

The versatility of these materials in automotive applications has been demonstrated through diverse studies. Negative Poisson’s ratio honeycomb structures were developed [79] using 3D-printed CFRCs, exhibiting lateral expansion under compression for energy-absorbing automotive parts. A 3D printing assembly technique was proposed [174] for composites with advanced photocatalytic functions, primarily for environmental applications but adaptable to automotive surface functionalization like self-cleaning coatings. Additionally, novel continuous fiber-reinforced thermoset composite honeycomb sandwich structures were studied [175], where polymethacrylimide foam reinforcement enhanced compressive performance for lightweight body panels or chassis components.

Customization capabilities extend to manufacturing lightweight body parts and specialized fixtures. In situ and adhesive repair techniques for CFRCs were proposed [176], enabling rapid automotive component restoration to optimize manufacturing and maintenance workflows. However, widespread automotive adoption requires addressing cost challenges and mass production feasibility, necessitating future research on process optimization and material recyclability.

### 6.3. Biomedical Applications

In the biomedical field, although research remains limited, 3D-printed CFRPs demonstrate emerging potential. As shown in Figure 5c, heterogeneous scaffolds fabricated via ultrafine fiber 3D printing were investigated [177], where optimized porosity and fiber alignment supported cell proliferation and differentiation for tissue engineering and regenerative medicine. The drop-weight impact performance of 3D-printed continuous carbon fiber-reinforced honeycomb structures was studied [178]; while focused on mechanical properties, their bio-inspired designs (e.g., mimicking owl feather barbule structures) may inspire prosthetic or biomedical device development.

Further research has broadened biomedical applications. A 3D printing technique for continuous aramid fiber composites was developed [179], creating beetle mandible-inspired structures with elongated fiber bundles for durable prosthetics or bone supports. Additionally, recoverable honeycomb composites from study [128] could enable dynamically adaptive implants like adjustable scaffolds or orthoses due to their shape recovery capabilities.

Nevertheless, the limited biocompatibility of carbon and glass fibers restricts direct biomedical use. Studies [178,179] suggest future directions may involve exploring biocompatible fibers (e.g., natural fibers) or surface modification techniques. For instance, the heterogeneous scaffold design from [177] could integrate biocompatible matrices to advance tissue engineering applications. Overall, biomedical research remains nascent, requiring interdisciplinary collaboration to develop clinically viable materials.

### 6.4. Civil Engineering Application

In recent years, the principles of green building and sustainable development have driven researchers in civil engineering to focus on 3D printing construction technologies. This approach enables rapid customization of buildings and structural components while eliminating the additional costs and labor associated with formwork fabrication, establishing 3D printing as one of the most promising directions in the contemporary construction industry [180].

Concrete remains the primary material for this technology, with fiber-reinforced polymer (FRP) composites being proposed as alternatives to conventional steel reinforcement for strengthening 3D-printed concrete structures. Numerous reinforcement methods for 3D-printed concrete have recently been developed and implemented [181]. For instance, Fan et al. [182] investigated the effects of polyethylene and steel fibers on the flexural performance of FRP-reinforced ultra-high-performance concrete (UHPC) tubular beams under static and fatigue loading. The results demonstrated that the steel fiber-reinforced beams exhibited higher load-carrying capacity under static loading, with a peak load 34.5% greater than polyethylene-reinforced beams. However, under equivalent fatigue loading conditions, the steel fiber-reinforced specimens showed faster degradation in the UHPC compression zone. Zeng’s team [183] investigated the effects of concrete strength, fiber volume fraction, anchorage length, and fabrication process on the bonding performance between GFRP bars and 3D-printed concrete (3DPC) through pull-out tests and validated the applicability of conventional bond-slip models for FRP-reinforced 3DPC. The results indicate that bar direction and fiber volume fraction have a significant impact on bond strength, whereas anchorage length and concrete strength exert a more moderate effect. Yan et al. [184] proposed using FRP wraps to further enhance the compressive performance of 3D-printed ultra-high-performance concrete (3DPU) components. Through axial compression tests examining different loading directions and FRP confinement thicknesses (0, 1, and 2 layers), their findings revealed that FRP wrapping significantly improved both the strength and deformability of 3DPU.

With the continuous development and innovation in fiber-reinforced polymer (FRP) bar production technology, thermoplastic FRP bars are gaining increasing popularity and are being more widely adopted as replacements for conventional steel reinforcement in concrete structures [185]. In the future, continuous fiber 3D printing technology is expected to establish increasingly stronger connections with civil engineering applications. However, several challenges persist. For example, conventional FRP bent bars are typically fabricated by bending straight bars, resulting in significantly reduced strength compared to straight reinforcement and consequently leading to premature failure at curved sections. In recent years, additive manufacturing-based continuous fiber-reinforced thermoplastic composites (AM-CFRTPs) have undergone rapid development. These mate-rials offer superior mechanical properties, ease of fabrication, and excellent in situ formability. Most importantly, they enable rapid production of complex geometries while avoiding excessive compression or tension during manufacturing. As such, AM-CFRTP-based reinforcements show great potential to replace conventional FRP and steel reinforcements in construction, providing an effective solution to the challenges posed by complex-shaped reinforced structures [186].

### 6.5. Other Applications

Beyond the primary domains discussed, 3D-printed CFRPs demonstrate potential in additional fields. The photocatalytic composites from [174], initially designed for environmental remediation, could be adapted for energy applications such as high-efficiency solar energy harvesters. Bio-inspired design concepts from [170,171] might be extended to robotics for manufacturing lightweight yet durable robotic components. The sandwich structures investigated in [111], recognized for their high stiffness and load-bearing capacity, could also be applied to lightweight panels in construction or marine industries.

In summary, the application domains of 3D-printed CFRPs are rapidly expanding—from traditional engineering sectors like aerospace and automotive to exploratory fields such as biomedical and emerging technologies. By integrating topology optimization, bio-inspired designs, and functional enhancements, these materials provide innovative solutions across multiple industries. However, domain-specific challenges—including environmental durability in aerospace, cost-effectiveness in automotive, and biocompatibility in biomedical applications—require further research and resolution.

## 7. Challenges and Future Potential

Continuous fiber 3D printing technology has achieved remarkable progress in recent years, yet several critical challenges remain. From a mechanical performance perspective, inadequate fiber–matrix interfacial bonding strength continues to be a significant weakness that compromises interlayer properties. Additionally, the anisotropic nature of printed structures, caused by alternating heating and cooling cycles during fabrication coupled with void formation, presents another prominent issue that limits the mechanical performance of printed components. Regarding process scalability, current printing speeds typically below 500 mm/s fail to meet industrial production requirements. For large-scale prints exceeding 1 cubic meter, precise control of fiber orientation and equipment stability remain technical bottlenecks. Equipment limitations also constrain fiber volume fraction below 50% in most commercially available 3D printers, which is insufficient for extreme service conditions, while nozzle clogging and other reliability issues require ongoing optimization. Material-wise, there remains a shortage of resin systems suitable for extreme temperature environments.

Despite these challenges, continuous fiber 3D printing has demonstrated expanding application potential. Researchers are actively working to improve the technology through various approaches, including optimization of printing paths, development of hybrid fiber systems, and integration with artificial intelligence, aiming to reduce the limitations compared to conventional manufacturing methods.

## 8. Conclusions

3D printing technology for CFRPs has established novel pathways for manufacturing high-performance composites through synergistic innovation in material selection, process optimization, and mechanical-functional integration. The selection of fiber–matrix systems forms the core of this technology: carbon fibers dominate aerospace applications due to their exceptional strength-to-weight ratios, glass fibers offer economic advantages, while natural fibers exhibit potential for sustainable applications. Thermoplastic matrices like PEEK, processed via high-temperature extrusion, remain the mainstream choice for complex structural fabrication. At the process level, FDM ensures robust fiber–matrix interfaces through pre-impregnated and in situ impregnation strategies, whereas photopolymerization techniques theoretically provide higher precision but face challenges in fiber alignment within liquid resins.

The customization of mechanical properties originates from coupled multi-factor mechanisms. Fiber type defines performance baselines, while volume fraction and orientation distributions regulate strength, stiffness, and toughness via topology optimization. Printing parameters and post-processing techniques enhance overall performance through micro-interfacial and macro-structural modifications. Functional integration—such as electrical conductivity, thermal management, self-sensing, and shape memory behaviors—primarily derives from specialized fiber types. Consequently, concurrent integration of functionality and mechanical performance emerges as a critical research frontier, with future goals focusing on achieving mechanical-functional synergy while maintaining functional capabilities.

The ultimate objective of mechanical-functional integration lies in application-specific adaptation. Examples include designing lightweight high-performance aerospace components, recoverable honeycomb structures, or multi-fiber hybrid crash-absorbing parts for automotive applications, synergizing lightweighting and impact resistance.

Despite challenges like cost control and long-term environmental stability—and while absolute mechanical performance still lags behind traditional continuous fiber composites—the superior design flexibility, rapid prototyping, and precise fiber path control of 3D printing technologies unlock unprecedented application potential. Breakthroughs in hybrid material systems and multi-axis printing continue to expand application boundaries. Future integration of artificial intelligence (AI) and 3D printing is poised to accelerate process optimization, driving this technology from laboratory innovation to industrial-scale manufacturing.

## Figures and Tables

**Table 1:** Fibers for 3D-printed CFRPs.

Property	Carbon Fiber	Glass Fiber	Aramid Fiber	Natural Fiber	Hybrid Fiber
**Characteristics**	High strength-to-weight ratio, High modulus, Corrosion resistance	Good mechanical properties, Low cost, High toughness	High strength, High modulus, Heat resistance	Sustainability, Biodegradability, Low density	Performance optimization, Cost-performance balance
**Density** **(g/cm^3^)**	1.8	2.5	1.44		
**Tensile strength (Mpa)**	3500–7000	2000–4000	2800–4100		
**Modulus (GPa)**	230–600	70–90	60–130		
**References**	[8,9,10,11,12,13,14,15]	[16,17,18,19]	[20,21]	[22,23,24,25,26,27,28]	[19,21]

**Table 2:** Matrix material for 3D-printed CFRPs.

Matrix Material	Type	Characteristics	Application Fields	References
**PLA**	Thermoplastic	Biodegradability, Low cost, Easy processing; Lower mechanical properties and thermal stability (softens at 50–60 °C)	Beginner projects, Biomedical applications	[14,22,26]
**PA6**		Excellent mechanical properties, High-temperature stability	Aerospace, Automotive industry	[10,13]
**PEEK**		High continuous service temperature (250 °C), Outstanding chemical stability, High mechanical performance	Aerospace, Automotive industry, Extreme environments	[11,30,31,32,33]
**ABS**		Good impact resistance, excellent electrical insulation properties, superior chemical stability	Electrical Engineering, Functional Prototyping	[34]
**Epoxy resin**	Thermosetting	High insulation properties, Excellent mechanical performance, High moisture resistance; Complex curing process, Relatively low flame resistance	Aerospace, Automotive industry, Electronics and electrical engineering	[8]
**Phenolic resin**		High temperature resistance, Excellent chemical stability, Short curing time; High water absorption, Low toughness	Fire-resistant materials, Acid and alkali-resistant chemical equipment	[35]

**Table 4:** The effects of key process parameters in 3D printing of CFRPs.

Parameter	Effects	Optimization Methods	References
**Printing speed**	Affects fiber placement accuracy and matrix distribution; High speed may increase porosity	Adjust speed to balance precision and efficiency	[17,68,83,84]
**Nozzle ** **temperature**	Excessive temperature may degrade matrix; Insufficient temperature leads to poor impregnation	Adaptive temperature control based on material properties	[85,86,87,88]
**Fiber feed rate**	Mismatch with extrusion rate causes fiber accumulation or matrix deficiency	Synchronize feed and extrusion rates to maintain consistent fiber volume fraction	[89,90,91]
**Path ** **planning**	Determines fiber orientation and part anisotropy	Load-dependent path planning or geometry-driven path optimization	[92,93,94,95,96,97]

**Table 5:** Summary of factors influencing the mechanical properties of 3D-printed CFRPs.

Influencing Factor	Affected Mechanical Properties	Optimization Strategies	References
**Fiber type**	Tensile strength, Compressive strength, Flexural modulus, Impact toughness	Select carbon fibers (high strength), glass fibers (cost-effective), or aramid fibers (toughness)	[99,100,101,102,103]
**Fiber volume fraction**	Tensile strength, Compressive strength, Flexural modulus, Interlaminar bonding strength	Maintain 40–50% volume fraction to balance performance and porosity	[54,104,105,106,107,108]
**Printing ** **parameters**	Tensile strength, Compressive strength, Flexural modulus, Impact toughness, Interlaminar bonding strength	Adjust nozzle temperature (avoid degradation), optimize printing speed (balance efficiency/quality), modify layer thickness (enhance interlayer bonding)	[85,105,109,110,111,112,113,114,115,116,117]
**Fiber ** **orientation**	Tensile strength, Compressive strength, Flexural modulus, Impact toughness, Interlaminar bonding strength	Align fibers along load direction, topology-optimized paths, hybrid fiber layouts	[118,119,120,121,122,123,124,125,126,127,128,129]
**Interfacial ** **issues**	Shear strength, Tensile strength, Flexural strength	Modification of interlayer stacking sequence, Adjustment of fiber orientation, Application of composite fiber interfaces	[130,131,132,133,134,135,136]
**Fiber ** **pretreatment**	Tensile strength, Interfacial adhesion, Overall reliability	Surface coating, chemical modification	[137,138,139,140,141,142,143,144,145]
**Structural ** **design**	Tensile strength, Flexural modulus, Impact toughness, Energy absorption efficiency	Topology optimization, honeycomb structures, multi-scale optimization	[146,147,148,149]
**Hybrid fiber systems and ** **advanced ** **processes**	Tensile strength, Compressive strength, Flexural modulus, Impact toughness	Carbon/glass or carbon/aramid fiber hybrids, multi-axial alignment	[150,151,152,153,154,155,156,157,158]

## Data Availability

Data available on request.
